# Anesthesia Depresses Cerebrovascular Reactivity to Acetazolamide in Pediatric Moyamoya Vasculopathy

**DOI:** 10.3390/jcm12134393

**Published:** 2023-06-29

**Authors:** Pieter T. Deckers, Jeroen C. W. Siero, Maarten O. Mensink, Annick Kronenburg, Kees P. J. Braun, Albert van der Zwan, Alex A. Bhogal

**Affiliations:** 1 Department of Neurosurgery, Universitair Medisch Centrum Utrecht, 3584 CX Utrecht, The Netherlands; a.vanderzwan@umcutrecht.nl; 2 Department of Radiology and Nuclear Medicine, Meander Medisch Centrum, 3813 TZ Amersfoort, The Netherlands; 3 Department of Radiology, Universitair Medisch Centrum Utrecht, 3584 CX Utrecht, The Netherlands; j.c.w.siero@umcutrecht.nl (J.C.W.S.); a.bhogal@umcutrecht.nl (A.A.B.); 4 Spinoza Center for Neuroimaging, 1105 BK Amsterdam, The Netherlands; 5 Pediatric Anesthesiology, Prinses Máxima Centrum, 3584 CS Utrecht, The Netherlands; m.o.mensink-3@umcutrecht.nl; 6 Department of Neurosurgery, Leiden University Medical Center, 2333 ZA Leiden, The Netherlands; a.kronenburg@umcutrecht.nl; 7 Department of Neurosurgery, Haaglanden Medical Center, 2512 VA The Hague, The Netherlands; 8 Department of Pediatric Neurology, Wilhelmina Children’s Hospital, Universitair Medisch Centrum Utrecht, 3584 CX Utrecht, The Netherlands; k.braun@umcutrecht.nl

**Keywords:** cerebrovascular reactivity, moyamoya, anesthesia, BOLD, ASL, acetazolamide

## Abstract

Measurements of cerebrovascular reactivity (CVR) are essential for treatment decisions in moyamoya vasculopathy (MMV). Since MMV patients are often young or cognitively impaired, anesthesia is commonly used to limit motion artifacts. Our aim was to investigate the effect of anesthesia on the CVR in pediatric MMV. We compared the CVR with multidelay-ASL and BOLD MRI, using acetazolamide as a vascular stimulus, in all awake and anesthesia pediatric MMV scans at our institution. Since a heterogeneity in disease and treatment influences the CVR, we focused on the (unaffected) cerebellum. Ten awake and nine anesthetized patients were included. The post-acetazolamide CBF and ASL-CVR were significantly lower in anesthesia patients (47.1 ± 15.4 vs. 61.4 ± 12.1, *p* = 0.04; 12.3 ± 8.4 vs. 23.7 ± 12.2 mL/100 g/min, *p* = 0.03, respectively). The final BOLD-CVR increase (0.39 ± 0.58 vs. 3.6 ± 1.2% BOLD-change (mean/SD), *p* < 0.0001), maximum slope of increase (0.0050 ± 0.0040%/s vs. 0.017 ± 0.0059%, *p* < 0.0001), and time to maximum BOLD-increase (~463 ± 136 and ~697 ± 144 s, *p* = 0.0028) were all significantly lower in the anesthesia group. We conclude that the response to acetazolamide is distinctively different between awake and anesthetized MMV patients, and we hypothesize that these findings can also apply to other diseases and methods of measuring CVR under anesthesia. Considering that treatment decisions heavily depend on CVR status, caution is warranted when assessing CVR under anesthesia.

## 1. Introduction

Cerebrovascular reactivity (CVR) measurements are increasingly used to provide diagnostic information and to aid treatment decisions in a range of cerebrovascular diseases, including moyamoya vasculopathy (MMV) [1,2,3]. While [^15^O]H_2_O-PET has long been considered the gold standard, non-invasive MRI-based measurements, such as Arterial Spin Labeling (ASL), are increasingly employed in clinical practice [4,5,6,7]. In order to probe vascular reserve capacity, vasoactive stimuli such as intravenous acetazolamide or the inhalation of CO_2_-rich air are required [8,9]. CVR can then be calculated by subtracting perfusion scans acquired during the stimulus from those acquired under baseline conditions, a similar process as with [^15^O]H_2_O-PET. Brain regions with low or absent CVR, or even steal (a regional paradoxical decrease in CBF after the stimulus), are associated with a higher infarction risk, and therefore warrant treatment. For MMV, this involves surgical revascularization [4,10,11]. An important consideration for these patient groups is that vascular occlusion and significant collateralization can lead to long blood transit times that can be problematic for standard single-time-point ASL protocols. The short half-life (<2 s at 3T) of the endogenous magnetic label results in arterial transit artifacts and an artificially low tissue perfusion signal. Fortunately, this can be mitigated using Multidelay-ASL (MD-ASL) strategies with kinetic modeling to improve the accuracy of CBF quantification in such cases [5,7].

Since ASL is a subtraction-based technique, it suffers from an inherently low signal-to-noise ratio (SNR) and, in order to acquire high-quality perfusion images, relatively long scan-times of the order or 4–5 min are required. As a consequence, ASL is not a suitable method for accurate measurement of dynamic flow responses. An alternative technique is blood-oxygenation-level-dependent (BOLD) imaging, in which signal contrast is dependent on the total amount of deoxygenated blood within a voxel. This BOLD-signal can be modulated by changes in CBF but also by cerebral blood volume (CBV), the cerebral metabolic rate of oxygen consumption (CMRO_2_), body temperature, oxygenation level, and metal remnants (i.e., surgical implants) [12,13,14,15,16]. Major benefits of BOLD MRI are the high SNR (allowing for higher image resolution compared to ASL) and short repetition time (TR of 1–3 s), facilitating the tracking of hemodynamics. When used for CVR-measurements, the BOLD-signal reflects the alterations in venous oxygen saturation, caused by CVR-mediated changes in CBF. As a result, BOLD-CVR responses are often considered as surrogates for changes in CBF [4,16,17]. In the current work, we exploit these properties to investigate the dynamic vascular responses in the brain caused by acetazolamide (ACS) injection. 

The time-course of the ACZ response generally reflects maximum vasodilation around 12–15 min after injection; however, this can vary between individuals and/or brain regions [18,19,20]. Taking this into consideration, a full survey of quantitative and dynamic flow responses necessitates—in addition to standard clinical imaging—a long examination time. Since MMV patients are often young or cognitively impaired, the administration of anesthesia is often required to prevent anxiety- and motion-related artifacts [21,22,23]. Despite the diagnostic significance of hemodynamic parameters derived from BOLD and ASL imaging in pediatric MMV, reports on the effects of anesthesia on these parameters during ACZ administration are limited. Some focus has been placed on the effect of different anesthetic agents on CBF (e.g., using propofol versus sevoflurane anesthesia [24]), but ACZ is most often used according to the same protocol as in awake patients [25,26,27]. This is performed under the assumption that mechanisms of CVR are unaffected by the anesthetic agent. As the determination of CVR and steal is influenced by further (operative) treatment decisions, this assumption should be validated. Overestimation of the true CVR due to anesthesia effects could lead to under-treatment, which could ultimately result in preventable new infarctions or cognitive deterioration, while underestimation could lead to over-treatment, possibly resulting in unnecessary surgical risks [28].

To investigate the effect of anesthesia on the CVR, we compared the response to ACZ between awake and anesthetized patients using MD-ASL before and ~15 min after injection of ACZ, combined with a dynamic BOLD series, to benefit from both techniques while gaining valuable insights into the dynamic effect of ACZ on the cerebrovascular hemodynamics. Since the MMV-pathology can have heterogeneous spatial effects in the brain, including widespread vascular steal, we chose the cerebellum—the brain region least affected by MMV and often used as a reference area for the effect of the stimulus—to assess the effect of anesthesia on CVR. We hypothesized that the general clinical assumption of a limited influence of anesthesia on CVR is false, and that CVR-maps acquired under anesthesia differ from those in awake patients. 

## 2. Materials and Methods

This study was submitted to the Medical Ethics Review Committee of the UMC Utrecht, which confirmed that the Medical Research Involving Human Subjects Act (WMO) did not apply. All patients or parents provided informed consent. Our institute implemented a new MRI protocol for clinical CVR measurements using ACZ since 2018, as an alternative to PET scans, for which patients would have to be referred to another hospital. We retrospectively reviewed our clinical database and included all pediatric MMV patients (proven by angiography or MRA, using the standardized criteria of the Research Committee on the Pathology and Treatment of Spontaneous Occlusion of the Circle of Willis [29]) scanned both awake and under anesthesia. Imaging data that were corrupted by artifacts (i.e., motion, extreme distortion due to surgical clips) were excluded. For patients scanned at multiple time-points, the first (usually preoperative) scan was included. 

### 2.1. Anesthesia

All patients were screened by a specialized pediatric anesthesiologist prior to the investigation. Anesthesia was induced with either propofol or sevoflurane, and maintained with either propofol (*n* = 4) or sevoflurane (*n* = 5), at the discretion of the anesthesiologist and after consultation with patient and parents. Maintenance of anesthesia during the ACZ challenge requires a second IV-drip, which was a factor for choosing sevoflurane in some patients. Breathing was managed with a laryngeal mask. No muscle relaxants were administered, so the breathing of the patients was self-paced. During anesthesia, blood pressure was measured non-invasively every three minutes; oxygen saturation, heart rate, respiratory rate, and pEtCO_2_ were measured every minute; and the rate of added O_2_ to inspiratory air was monitored continuously, which is a standard clinical procedure during anesthesia in our center. If the blood pressure dropped below the pre-procedural set threshold, phenylephrine was administered intravenously. Vital parameters were not monitored during the standard MRI procedures in awake patients.

### 2.2. Scanning Protocol and Parameters

**Figure 1:**
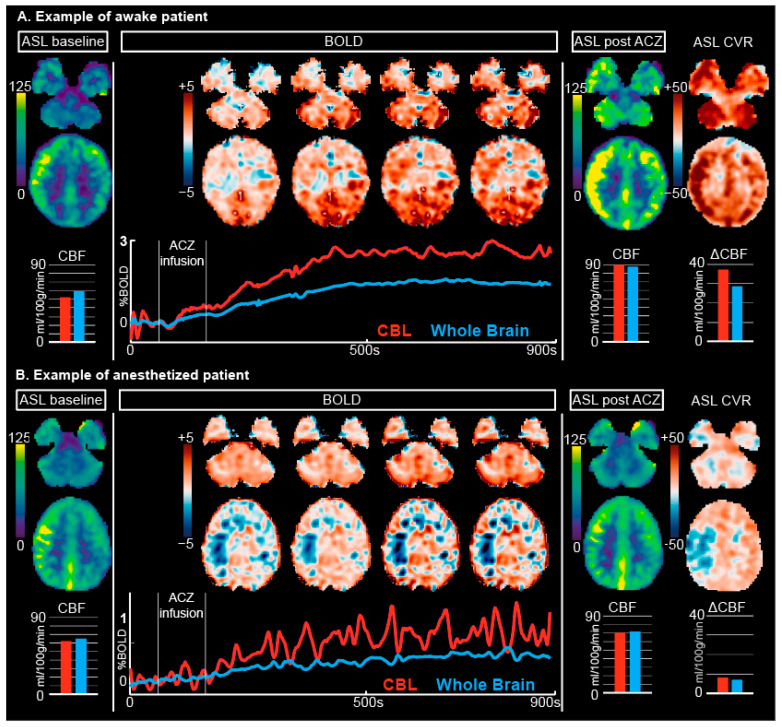
Example of protocol with awake ((**A**), age 15.6 years) and anesthetized ((**B**), age 9.6 years, sevoflurane) patient. The protocol is shown from left to right, with the calculated ASL-CVR on the far right, based on the pre- and post-ACZ scans, and expressed as change in mL/100 g/min. The results of the scan are shown in two slices (upper mainly cerebellar, lower supratentorial). For the BOLD scan, the gradual increase in the BOLD signal (in %) over time is shown. Note the difference in range of the *y*-axis of the BOLD signal between the awake patient (from 0–3%) and anesthetized patient (from 0–1%). Red: cerebellum (CBL), Blue: whole brain.

All scans were performed on a 3T MRI system (Philips, Best, The Netherlands) using a 32-channel receive array (Nova Medical, Inc., Wilmington, MA, USA). Baseline CBF measurements were obtained with MD-ASL: 5 post-labeling delays (1206–3480 ms), pseudocontinuous ASL (pCASL), multi-slice Echo Planar Imaging (EPI), label duration = 2 s, voxel-size = 3.75 × 3.75 × 7 mm^3^, 16 slices, Field of View (FOV) = 240 × 240 × 120 mm^3^, TR/TE = 6 s/11 ms, flip angle = 25°, SENSitivity Encoding (SENSE) factor = 2, 4 background suppression, 24 volumes, scan-time = 5 min. The ASL was planned using a phase contrast angiography scan, with the labeling plane placed perpendicular to the internal carotid arteries and vertebral arteries. A 15 min multi-slice gradient-echo EPI (BOLD) scan was acquired with the following parameters: voxel size = 2.5 mm isotropic, 48 slices, FOV = 224 × 224 × 120 mm^3^, TR = 1.1 s (multiband, *n* = 12) or TR = 2.8 s (non-multiband, *n* = 7), TE = 35 ms, flip angle = 65° SENSE factor = 1.7, scan time = 15.5 min. The ACZ injection (20 mg/kg (maximum 1 g) in 30 cc of 0.9%NaCl, flowrate of ~0.3 cc/s) began between 60 and 90 s after the start of the BOLD sequence. Upon completion of the BOLD scan, a second MD-ASL scan with identical parameters as the baseline scan was acquired and used for the calculation of the ASL-CVR (Figure 1). Finally, a 3D-T1 anatomical scan was performed for spatial normalization, and depending on the clinical question, most patients received additional T2-flair, SWI, MRA, or other standard clinical scans (not used in this investigation). 

The ASL data were analyzed with an in-house-developed pipeline script in MATLAB (version: 9.10.0 (R2021a); Mathworks, Natick, MA, USA), and consisted of making a T1-weighted image from the multiple PLD M0 images, of both the pre- and post-ACZ scans, to segment the scans into white matter (WM), grey matter (GM), and cerebral spinal fluid (CSF) and to register the post-ACZ scan to the pre-ACZ scan. Outlier removal was performed, based on the standard deviation and tissue variance [30]. Quantitative CBF maps were generated using the BASIL tool (FSL) and a subtraction image for the CVR was computed. 

BOLD data were motion-corrected (MCFLIRT [31]; FMRIB Software Library [32] (FSL, Oxford, UK)), distortion-corrected (TOPUP, FSL; including the use of the test-BOLD sequence), spatially smoothed (2D Gaussian kernel, FWHM = 5 mm), and—using the baseline period before ACZ injection—converted to %ΔBOLD. Large vessel signals were removed and wavelet-based temporal de-noising was applied (seeVR, Utrecht, The Netherlands) [33]. Considering the different scan TRs used in this cohort, all BOLD data were interpolated to TR = 1 s. Since ACZ induced a very gradual increase, the ‘sampling rates’ of both TRs (1.1 and 2.8 s; on a scan of ~930 s) were high enough for reliable interpolation. Furthermore, the data were smoothed and longer trends were visualized, so the different TRs had no effect on the analysis or group comparisons. 

#### Regions of Interest, Data Processing, and Statistics

The MNI 1 mm brain was linearly registered to the patient-specific T1 scan using the Linear Image Registration Tool (FLIRT; FSL) [31]. The T1 was registered to both the mean-BOLD and the T1-M0 image of the ASL using FLIRT. After concatenating the transformation matrices of MNI to T1, and T1 to ASL and BOLD space, the cerebellar mask from the MNI structural atlas was transformed to BOLD and ASL [34,35]. All registration steps were carefully checked visually. The cerebellar masks were applied to generate 1D BOLD-CVR response time series. 

For ASL, the cerebellum mask was used to extract the mean CBF values from the individual CBF and CVR maps using FSL, and these values were visualized in a boxplot and compared using Student’s *t*-test. For the BOLD-CVR response, the mean and 95%CI (1.96*SEM) were plotted for the awake and anesthesia group. 

For every individual patient, the BOLD signal was further temporally smoothed (LOESS filter, 6% regression window) to calculate the final BOLD increase (defined as Δ%BOLD between baseline and highest value of the last 5 s of the scan), time to maximum BOLD signal, and the maximum slope of the initial linear response. These individual values were visualized with boxplots and compared between groups using Student’s *t*-test (MATLAB). 

The recorded vital parameters (pEtCO_2_, heart rate, breathing rate, mean arterial pressure (MAP)) of the anesthesia group were normalized to the two minutes before the start of the ACZ challenge so the changes could be expressed in percentages, and were plotted with 95%CI. The individual percentage of 100% oxygen administered to the inspiratory air of the anesthesia patients was graphically compared with the maximum BOLD response in a scatter plot (e.g., 10% corresponds to an inspiratory air mixture of 10% pure oxygen and 90% room-air). The correlation between added oxygen and maximum BOLD response was calculated with Pearson’s correlation test. The differences between propofol and sevoflurane were compared using a Student’s *t*-test. For all tests, a *p*-value of <0.05 was considered significant. All values were checked for normality visually and formally using the Kolmogorov–Smirnov test. 

## 3. Results

Twenty-three datasets of unique pediatric patients were identified. Two datasets (one anesthesia and one awake) could not be used, due to severe motion or metallic-surgical-remnants-related artifacts, and the two first (pilot-) datasets could not be used, due to the use of different scanning techniques. We included nine children scanned under anesthesia (four MMD, five MMS; two unilateral, seven female; median age (range) 11.5 (5.9–16.4)) and included ten awake children (eight MMD, two MMS; four unilateral, six female; age 13.4 (6.8–17.2)) for final analysis (Table 1). 

### 3.1. ASL

**Figure 2:**
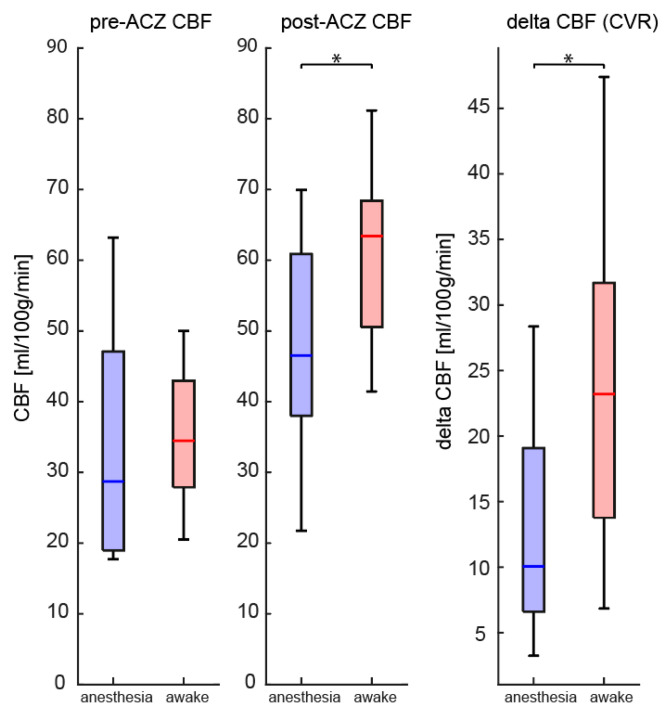
Arterial Spin Labeling CBF response to Acetazolamide (ACZ). Comparison of quantified cerebral blood flow (CBF) of the cerebellum between anesthetized (*n* = 9) and awake (*n* = 10) patients, measured by multidelay arterial spin labeling, before and after ACZ injection, and the cerebrovascular reactivity (CVR, expressed as Δ-CBF). *: *p* < 0.05.

The baseline ASL-CBF values in the cerebellum were comparable between anesthetized and awake patients (34.9 ± 17.2; 36.0 ± 9.4 mL/100 g/min, *p* = 0.86, respectively, Figure 1 and Figure 2). The ASL-CBF values fifteen minutes after ACZ-injection of the awake patients had both significantly higher CBF (61.4 ± 12.1 vs. 47.1 ± 15.4, *p* = 0.037) and CVR, expressed as ΔCBF (23.7 ± 12.2 vs. 13.3 ± 8.4 mL/100 g/min, *p* = 0.031, Figure 2), compared to patients under anesthesia.

### 3.2. BOLD

**Figure 3:**
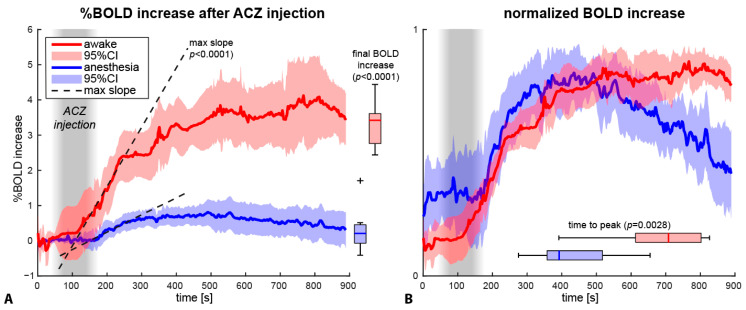
Mean time series of the BOLD-response of the (unaffected) cerebellum in awake patients (red) and anesthetized patients (blue). (**A**): %BOLD-change. (**B**): Normalized from minimum (0) to maximum BOLD-response (1), to compare the peak signal. The curves show the mean of the group data with a 95% confidence interval. Three metrics are compared: the maximum (max) slope (group average for both groups is shown by dotted lines), final BOLD increase (vertical boxplots, (**A**)), and the time to peak (horizontal boxplots, (**B**)).

The BOLD responses to ACZ on visual inspection were clearly different between scanned individuals who were awake and who were under anesthesia (Figure 1 and Figure 3). The group-averaged dynamic BOLD responses in the cerebellum became significantly different between awake and anesthesia patients ~120 s after starting the ACZ injection (Figure 3). The mean time to the maximum BOLD signal was ~463 ± 136 s (mean/SD) and ~697 ± 144 s in anesthetized and awake patients, respectively (*p* = 0.0035, Figure 3b). The final BOLD-CVR increase (i.e., the difference between baseline and the maximum BOLD signal of the last 5 s) was 0.39 ± 0.58% for anesthetized and 3.6 ± 1.2% for awake patients (*p* < 0.0001). The CVR-slope was 0.0050 ± 0.0040%/s and 0.017 ± 0.0059%/s for the anesthesia and awake patients, respectively (*p* < 0.0001, Figure 3a). 

### 3.3. Difference in Anesthesia Type 

**Figure A1:**
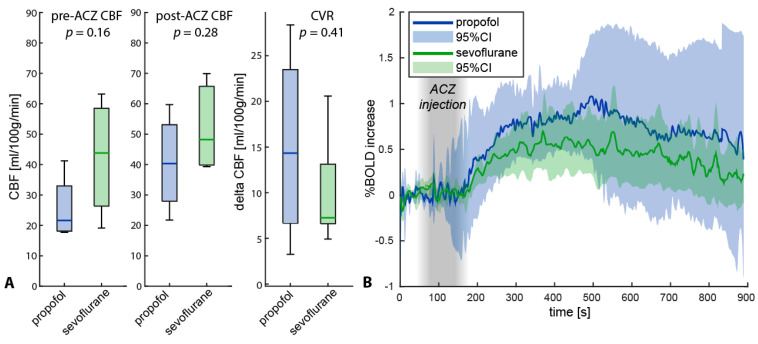
Difference in response between patients under anesthesia with propofol (blue, *n* = 4) and sevoflurane (green, *n* = 5). (**A**): Comparison of quantified cerebral blood flow (CBF) of the (unaffected) cerebellum, measured by multidelay arterial spin labeling, before and after ACZ injection, and the cerebrovascular reactivity (CVR, expressed as Δ-CBF). (**B**): Mean time series of the BOLD-response of the cerebellum.

When comparing the patients scanned during sevoflurane (*n* = 5) and propofol (*n* = 4) anesthesia, the ASL-CBF pre-ACZ was higher in the sevoflurane group (42.4 ± 18.5 vs. 25.5 ± 10.9 mL/100 g/min), while the CVR was lower (10.1 ± 6.2 vs. 15.1 ± 10.9 mL/100 g/min), although both these findings were non-significant (Appendix A: Figure A1A). The cerebellar BOLD-responses were slightly lower in patients using sevoflurane, but the difference was non-significant (within the 95%CI, Appendix A: Figure A1B). 

### 3.4. Vital Parameters of Anesthesia Patients

For the patients under anesthesia, the pEtCO_2_ clearly decreased after ACZ injection, while ACZ had less of an effect on the breathing rate, heart rate, and blood pressure. The lowest point (−18%) of the mean pEtCO_2_ was reached after ~8 min, after which it slowly rose again, which roughly inversely correlated with the time to maximum BOLD signal (Appendix B and Appendix C). A Pearson correlation coefficient was computed to assess the relationship between the final BOLD increase and the average concentration of added oxygen. There was a negative correlation between the two variables, *r*(*df*) = −0.698 (95%CI −0.931–−0.0638), *p* = 0.036). 

## 4. Discussion

Our primary research question was whether anesthesia has any effect on CVR measurements using ASL and BOLD imaging. We found an approximately two times lower ASL CBF increase in the unaffected cerebellum in anesthetized as compared to awake children with MMV. This effect was even stronger in the BOLD response, where the anesthesia group signal was characterized by a shorter time to reach the maximum BOLD-signal and a lower CVR-slope as well as an approximate fourfold lower maximum signal amplitude compared to awake patients. The main implication of this finding is that using these methods in combination with anesthesia may lead to an underestimation of the true CVR, although the effect on the presence and location of steal is still unclear. Since ASL provides quantitative flow values similar to [^15^O]H_2_O-PET, our results also imply that CVR may also be affected by anesthesia when using alternative CBF measurement techniques. Lower values found during anesthesia directly affect the CVR-maps used by clinicians, which may have implications for treatment strategies in MMV and other cerebrovascular diseases. 

To the best of our knowledge, the use of ACZ under anesthesia has not yet been systematically investigated, and the current literature describing CVR measurements under anesthesia for MMV is sparse, despite the fact that ACZ is one of the most commonly used vascular stimuli for probing CVR [8,9,18]. Venkatraghavan et al. published two feasibility studies investigating cerebral hemodynamics under anesthetized MMV patients. In the first, ASL-CBF (without an extra stimulus for CVR) was compared between propofol and sevoflurane in the same patients by switching medication during the scan, showing an increase in CBF during sevoflurane compared to propofol, due to the vasodilatory effect of sevoflurane—our data follow the same trend but do not reach statistical significance (Appendix A: Figure A1) [24]. The other was a feasibility study using a computerized gas blender for CO_2_-administration under propofol anesthesia, by manually ventilating the patients while measuring CVR with BOLD [36]. The CVR in the anesthetized patients was lower compared to healthy, awake volunteers, but cannot be directly compared, since both the disease and anesthesia can affect CVR (only the supratentorial regions were compared). Other studies included patients scanned under anesthesia, but did not provide quantifiable measures to compare the CVR of the awake and anesthesia patients [27,37]. Furthermore, there are papers describing CVR in pediatric MMV patients using sedatives instead of anesthesia [26], or the use of anesthesia is not mentioned in the paper. Even though it is unlikely that very young children (one to five years old) can go through an extensive imaging protocol without undergoing some form of anesthesia or sedation, comparing results from these previous studies with ours is complicated [25]. For CVR under anesthesia, it has been shown with mostly transcranial Doppler studies that the response to CO_2_ under anesthesia is still present, as described in a review including 38 studies with a wide range of patients (excluding those undergoing revascularization for occlusive cerebrovascular disease) [38]. Interestingly, the reported CO_2_ reactivity values in this study were higher with isoflurane (a potent vasodilator) compared to propofol. A blunted hypercapnia-induced CBF response was reported for high-concentration inhalation agents, which is in line with our results.

The mechanisms causing the differences between awake and anesthetized scan conditions are not directly clear based on the available literature, neither do we understand why the CVR response is much more blunted with BOLD (approximately 25% of awake values) than with ASL (approximately 50%). Nevertheless, we provide two possible explanations: 

### 4.1. Differences in Baseline Conditions between Anesthetized and Awake Patients

The baseline condition (before ACZ injection) is not the same for both groups, for which the BOLD-signal is most sensitive. How the BOLD signal arises is a complicated process and is influenced by many factors (as explained by the Davis model [13,39]; Appendix E). The various factors in anesthesia (difference in CBF, CMRO_2_, use of oxygen; positive expiratory pressure and tubes) lead to a higher baseline venous oxygenation compared to baseline, restricting the possible BOLD-increase after ACZ increase (see Appendix E for a more detailed explanation). ASL is less influenced by those factors at baseline (although Figure 2 does show a little lower CBF in the anesthesia group), and is therefore probably more accurate in reflecting the true effect of anesthesia on the CVR.

### 4.2. Differences in Response to ACZ

The autoregulatory systems are also affected by anesthetized patients, which could influence the response to ACZ. ACZ, a carbonic anhydrase inhibitor, influences many processes in the body, but for a vascular challenge, the most important effects are the lowering of the pH and the direct vasodilatory effect on the vessel wall. This effect can partly be mitigated by hyperventilation, raising the pH again after ACZ injection. In awake subjects, the maximum dilatory effect is reached after 10–15 min, while our anesthetized patients showed a minimum in pEtCO_2_ ~8 min, which roughly corresponds to a peak in BOLD-response. Therefore, it can be hypothesized that there was a difference in the autoregulation between awake and anesthesia patients. Furthermore, anesthesia can directly affect both CMRO_2_ and CBF, which can in turn both influence ASL and BOLD, possibly decreasing the maximum CVR. These mechanisms are further substantiated in Appendix E. Besides autoregulation, an important difference is the monitoring of the anesthesiologist. If during the challenge, the blood pressure drops too much, phenylephrine is administered (this was the case in six patients), possibly reducing the CBF response. For the awake subjects, the blood pressure was not measured during the scanning, and no extra medication was administered.

Considering all factors influencing the BOLD response, it may not be the most optimal option for measuring CVR under anesthesia. These factors include the sensitivity to, e.g., CMRO_2_, CBF_0_, Hb_0_, CBV_0_, the partially unknown effect of anesthesia on those factors, the sensitivity to added inspiratory oxygen, and the possible regional differences due to anesthesia. This holds for both BOLD CVR-scans using ACZ and hypercapnic stimuli. We would advise to combine BOLD under anesthesia with more independent and quantifiable measurements, like ASL or PET.

Since treatment decisions are often based on the presence and location of steal, an important unanswered question is whether the lower CVR we found in the anesthesia group translates to a lower threshold for steal detection (and consequently leads to more areas of steal), or actually shows less steal due to the blunted effect on the CVR. Furthermore, the regional effects of the anesthesia on the brain could also lead to different areas showing steal. To answer this, further studies should ideally focus on scanning the same patients both awake and under anesthesia, or comparing larger groups of randomized subjects. Another option would be to compare the unaffected, unilateral hemisphere in preoperative patients (although, due to the circle of Willis, the severity of the affected contralateral hemisphere might still influence the hemodynamics of the unaffected hemisphere [40]). Until all unanswered questions and influences of anesthesia on the cerebral hemodynamics on CVR scans are clear, caution is warranted in the interpretation of scan results under anesthesia.

#### Limitations

A major limitation of this study is the retrospective design, leading to inherently different patients in the anesthesia group compared to the awake group. However, baseline patient characteristics were comparable between groups, and we primarily assessed cerebellar CVR, a brain region that is not affected by MMV. Possible differences in severity of the supratentorial vasculature between groups might theoretically still influence the cerebellar hemodynamics due to, e.g., collaterals from the posterior cerebral artery (similar to the mechanism of contralateral improvement after treatment of a single hemisphere [40]). Due to the retrospective design, we did not continuously monitor the pEtCO_2_, saturation and heart rate, blood pressure, and breathing rates of the awake patients, e.g., the difference in hyperventilation between groups could not be investigated. Ideally, it would also be important to compare the blood gas values and blood-acidity between groups; however, for the young patients, this would be considered too invasive. Also due to the retrospective nature, the anesthesia parameters were less controlled than in an ideal prospective experiment, resulting in, e.g., a difference in medication and the use of oxygen and phenylephrine at the discretion of the responsible anesthesiologist. However, since MMV-patients inherently have a higher risk of cerebral ischemia and other anesthesia-related complications, we think that this is inevitable for the safety of the patients in clinical practice. Another major limitation is the group size, which is, considering the rarity of MMV, substantial but still small. While this group was large enough to show a significant difference, further subgroup analysis (e.g., between medication and MMD and MMS) was not possible. 

## 5. Conclusions

We found that the use of general anesthesia has a major effect on the measured CVR in the non-affected cerebellum of MMV patients, as reflected by a lower CBF increase after ACZ and a blunted total CVR for both ASL and BOLD, and an earlier peak in BOLD-increase. Therefore, CVR measurement under anesthesia may lead to the underestimation of true CVR. This underestimation can directly impact treatment strategies and may lead to surgical overtreatment. The differences were most pronounced in the BOLD-CVR measurements, but also hold for ASL and therefore possibly for other ways of measuring CBF and CVR. More research is needed for the implications of these findings, and to find the best way to measure CVR under anesthesia. Until then, results of CVR measurements under anesthesia need to be interpreted with caution. 

## Figures and Tables

**Table 1:** Characteristics of included patients.

		Anesthesia (*n* = 9)	Awake (*n* = 10)
age (median, range)		11.5 (5.9–16.4)	13.4 (6.8–17.2)
female		7 (78%)	6 (60%)
MMV type	MMD	4 (44%)	8 (80%)
	MMS	5 (55%)	2 (20%)
side	bilateral	7 (78%)	6 (60%)
	unilateral	2 (22%)	4 (40%)
treatment	preoperative	4 (44%)	7 (70%)
	unilateral operated	1 (11%)	1 (10%)
	bilaterally operated	4 (44%)	2 (20%)

Abbreviations: MMV = moyamoya vasculopathy, MMD = moyamoya disease (idiopathic), MMS = moyamoya syndrome.

**Figure A2:**
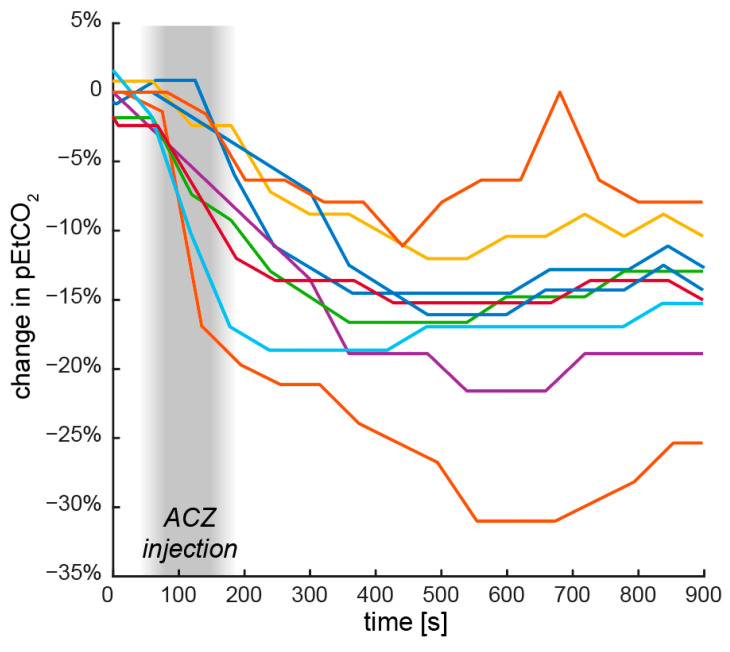
Percentage change in pEtCO_2_ after injection of acetazolamide of all individual pediatric anesthesia patients. The two minutes before acetazolamide injection were considered the baseline, and changes are expressed as percentages compared to the baseline.

**Figure A3:**
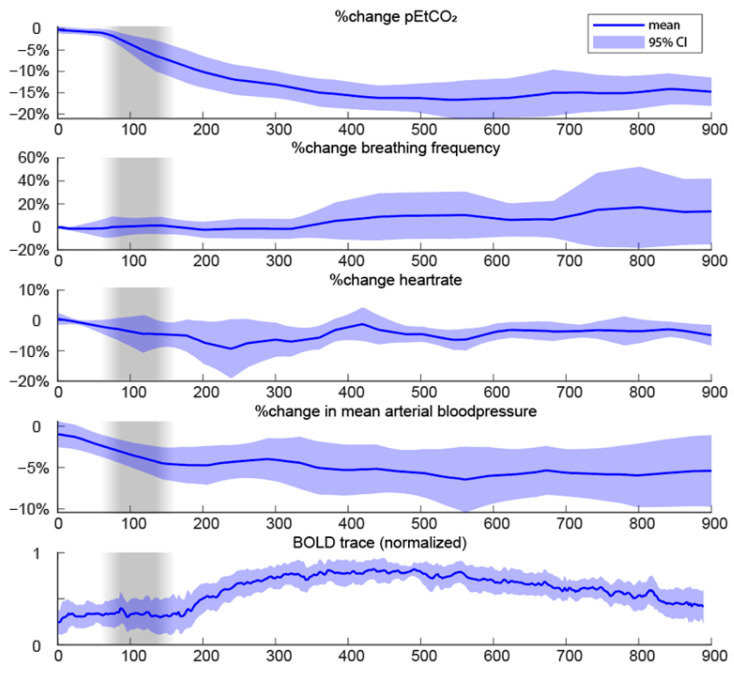
Physiological effects of ACZ on all pediatric patients under anesthesia. The two minutes before ACZ injection (between the vertical lines) were considered the baseline, and the measured values were expressed as the percentage change compared to baseline. The sampling rate of the pEtCO_2,_ breathing frequency, and heart rate was once every minute and that for mean arterial pressure was once every three minutes.

**Figure A4:**
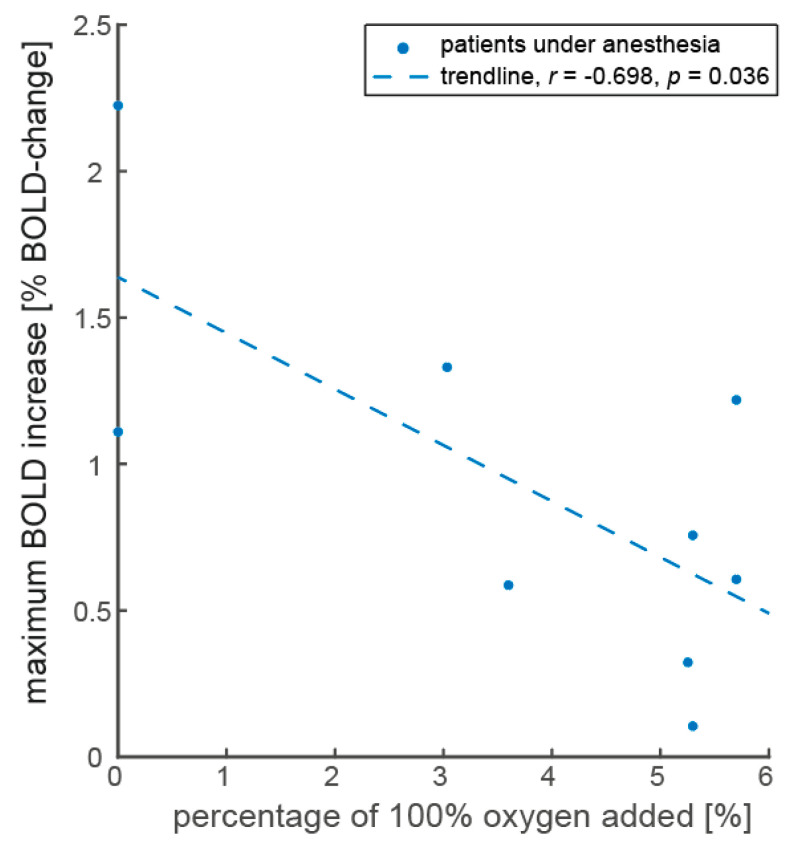
Scatterplot with trendline of %added oxygen and %maximum BOLD change and ASL-CVR. When deemed necessary by the responsible anesthesiologist, 100% O_2_ is added to the inspiratory air of the patient, e.g., 5% added O_2_ means the patient inspires 95% room air with 5% extra O_2_, resulting in a mixture of ~25% O_2_ and ~75%N_2_. An inverse relationship with the maximum BOLD-signal is shown by the trendline (*p* = 0.036).

## Data Availability

The data supporting this work are available upon reasonable request.

## Appendix E

### Appendix E.1. Differences in Baseline CBF between Anesthetized and Awake Condition

The baseline condition—before the injection of ACZ—is not completely the same for awake and anesthesia patients (although this is not clearly reflected by the baseline ASL-CBF values (Figure 2)). To understand the effect of the baseline differences on the BOLD-response, it is important to further understand the mechanisms driving the BOLD signal. As stated in the Davis model [13,39], the BOLD-signal change is mostly driven by changes in CBF, which is reflected in changes in venous deoxyhemoglobin, but also by the M-value and the CMRO_2_ change after the stimulus. It is likely that the various factors that changed during anesthesia (detailed below) lead to an increase in venous oxygenation, reducing the possible Δ-deoxyhemoglobin. The phenomenon of how changing the baseline condition before an experiment can change the BOLD response has been described previously; see, for example, Siero et al., who showed a reversal of neuronal-induced BOLD-response in 38% of patients after changing baseline conditions [20]; Halani et al. showing the BOLD and ASL response on three different levels of baseline pEtCO_2_ [41]; or Gauthier et al. showing elimination of visually evoked BOLD-responses after inhalation of Carbogen [42].

CMRO_2_ is reduced compared to awake patients by both propofol and sevoflurane, although propofol seems to decrease CMRO_2_ more than sevoflurane [43,44,45,46]. CMRO_2_ reduction does not immediately affect the ASL measurements, since the ASL signal is driven by a change in CBF. However, when CMRO_2_ decreases, while CBF remains the same, the ratio of oxygenated blood in the venous system increases at baseline, resulting in a lower possible BOLD response following a stimulus due to a lower M-value. Furthermore, it is good to consider that at baseline, the patients under anesthesia breathe through a laryngeal mask, tubes, and a machine. The anesthesia patients do not receive any muscle relaxants and the breathing is self-paced, but the added resistance will alter the breathing pattern. When deemed necessary by the anesthesiologist, positive expiratory pressure is applied during anesthesia, or oxygen is added to keep the arterial saturation high enough. Added oxygen can further increase the venous saturation at baseline, resulting in a less possible increase and therefore a lower maximum BOLD-response (Appendix D: Figure A4).

Anesthesia also affects baseline CBF. These effects need to be split between intravenous and volatile agents. Volatile agents, like sevoflurane, have a vasodilatory effect, leading to a direct increase in CBF [47]. Together with a decrease in CMRO_2_, this can be seen as an uncoupling of flow-metabolism matching [48]. This increase in CBF leads to an even higher venous saturation at baseline before the stimulus, consequently further lowering the possible BOLD signal increase to ACZ. Propofol reduces CBF (probably in response to the induced CMRO_2_ reduction, since isolated vessels in vitro have shown to dilate with propofol) [49,50]. Consequently, for propofol, there appears to be an intact CBF-CMRO_2_ coupling, contrary to sevoflurane, which is stronger than the direct vasodilatory effect on the vessels [48,51]. When comparing the baseline CBF values, measured by ASL, we do not see a statistically significant difference between awake and anesthesia patients. There is, however, a trend visible that propofol leads to a pre-ACZ CBF lower than that in awake patients, while inhalation anesthesia leads to a higher pre-ACZ CBF than awake patients, which is in line with the theory of the effect of anesthesia on the vasculature (Appendix A: Figure A1). Note that this effect of anesthesia on CBF has been used by Venkatraghavan et al. to show differences in CBF using sevoflurane and propofol in the same patient, as previously discussed [24].

All these baseline effects combined (the lowering of CMRO_2_, the change in CBF, and preoxygenation during anesthesia) lead to a higher venous saturation and lower possible Δ-deoxyhemoglobin (or reduction in the M-value), which results in a non-linear or asymptotic phase, where the same increase in CBF would lead to a much lower response of the BOLD signal (see Figure 1A in Hoge et al. [13]). We expect this higher venous oxygen at baseline to be the main reason that BOLD-CVR is reduced more compared to awake patients and to ASL-CVR.

### Appendix E.2. Differences in Response to ACZ and Autoregulation

Apart from the different baseline conditions due to anesthesia, the autoregulatory systems of the patients are also affected by the anesthetic state. This could influence the response to ACZ.

Normally, the vasodilatory effect of ACZ is caused by the inhibition of carbonic anhydrase, which is thought to affect the arterioles through two pathways: decreasing blood pH by an increase in H_2_CO_3_ and by a direct effect on the vessel wall [52,53]. Carbonic anhydrase is a catalytic enzyme found in the erythrocytes, where inhibition leads to an acidosis, which will lead to vasodilation. By inhibitions of the enzyme in the lungs, it decreases the expiratory pEtCO_2_ while raising the arterial pCO_2_ (and reducing the pH) [52,54]. These values normally closely correspond, but this coupling is disrupted by the blocking of carbonic anhydrase by the ACZ [55]. When carbonic anhydrase is blocked in walls of the cerebral arterioles, this could lead to a direct vasodilatory effect, although that has not been experimentally shown [9,56].

Directly after ACZ changes, the pH of the body will respond to restore the equilibrium based on the homeostasis principle. This secondary response leads to respiratory compensation of the acidosis by slight hyperventilation and deeper breathing [56]. This respiratory compensation increases blood pH and thus partly mitigates the effect of ACZ. Since patients can react differently, leading to a difference in total CBF change and time to maximum dilation, ACZ is not the perfect stimulus [9,18]. Most studies assume that the CBF increases to a plateau after around 10–15 min [9,18,25,57]. This has not been systematically researched for anesthesia patients before, but in our study, a peak is reached earlier (~7.7 min), without a lasting plateau phase. As seen in Appendix B and Appendix C, Figure A2 and Figure A3, only the pEtCO_2_ decreases significantly after ACZ injection, while the effect on breathing rate, heartrate, and blood pressure is not so clear. After a minimum at ~8 min, pEtCO_2_ starts to rise again. In healthy awake subjects, ventilation does increase after ACZ and the reduction in pEtCO_2_ has been shown to last longer [54,58]. This could point to a difference in the balance between the vasodilatory effect and the breathing rate increase between patients under anesthesia and awake, which could possibly explain the earlier reduction in the BOLD-signal in patients under anesthesia (Figure 3).

The effect of ACZ on CMRO_2_ is controversial, with some literature suggesting it to be isometabolic [59], while other papers report a decrease in CMRO_2_ [44]. To the best of our knowledge, it is still unknown what the effect is of ACZ on CMRO_2_ combined with anesthesia. Theoretically, ACZ could have an interaction with the anesthesia medication, causing a different response than in awake conditions. Since the mechanisms of action are profoundly different—anesthetics influence the GABA system, whereas ACZ affects carbonic anhydrase—it is unlikely this is the case. Since the BOLD signal in itself is non-quantitative, it is sensitive to a relative change in CMRO_2_ due to a stimulus (see the Davis model) [13,14]. Therefore, even if there is an effect on CMRO_2_ by ACZ and if that effect is similar between awake and anesthesia patients—i.e., yielding similar relative CMRO_2_ changes—this aspect is unlikely to play a role in the results we found. Anesthesia could, however, directly influence the vascular cells, reducing vasomotion and therefore reducing CVR [43]. Further research is needed to substantiate this possible effect and find the exact pathways leading to possible differences in autoregulation.
